# Osteopenia of prematurity and associated nutritional factors: case–control study

**DOI:** 10.1186/s12887-022-03581-y

**Published:** 2022-09-01

**Authors:** Mônica Raquel Chaves Pinto, Márcia Maria Tavares Machado, Daniela Vasconcelos de Azevedo, Luciano Lima Correia, Álvaro Jorge Madeiro Leite, Hermano Alexandre Lima Rocha

**Affiliations:** 1 Community Health Departament, Federal University of Ceará, Fortaleza, CE Brazil; 2 State University of Ceará, Fortaleza, CE Brazil; 3 Maternal and Child Health Departament, Federal University of Ceará, Fortaleza, CE Brazil; 4 Department of Global Health and Population, Harvard T. H. Chan School of Public Health, 677 Huntington Ave, Boston, MA 02115 USA

**Keywords:** Infant, Premature, Bone diseases, Metabolic, Nutritional support

## Abstract

**Background:**

Preterm newborn nutrition affects postnatal skeletal growth and bone mineralization, but studies have not yet fully concluded the relationship between nutrition and osteopenia. This study was intended to investigate the impact of nutritional factors on osteopenia in preterm newborns.

**Methods:**

This is a case–control study with babies born with gestational age ≤ 32 weeks in a high-risk maternity hospital, between 2018 and 2019. The population consisted of 115 newborns, being 46 cases (40%) and 69 controls (60%). Disease outcome was based on serum alkaline phosphatase levels > 900UL/l and hypophosphatemia < 4 mg/dl. Gestational data at birth and clinical and nutritional follow-up data during 8 weeks postnatally were assessed. Variables were assessed using regressive logistic models.

**Findings:**

Preterm infants who were fed pasteurized fresh human milk with acidity ≥ 4 ºDornic are 5.36 times more likely to develop osteopenia (*p* = 0.035). Higher calcium intake, compared to controls, also increased the probability of disease occurrence [OR 1.05 (CI 1.006–1.1); *p* = 0.025], while the presence of a partner [OR 0.10 (CI 0.02–0.59); *p* = 0.038] and the shortest time using sedatives [OR 0.89 (CI 0.83–0.98); *p* = 0.010] were protective factors associated with osteopenia. Extremely low birth weight [OR 5.49 (CI 1.20–25.1); *p* = 0.028], sepsis [OR 5.71 (CI 1.35–24.2); *p* = 0.018] and invasive ventilatory support [OR 1.09 (CI 1.03–1.18); *p* = 0.007] were risk factors.

**Conclusions:**

Acidity and high calcium intake are the main nutritional factors associated with osteopenia of prematurity. Further studies on the use of human milk with lower acidity, recommendation and nutritional supplementation of calcium should be accomplished to guide prevention strategies in newborns at risk for osteopenia during hospital stay.

## Background

Prematurity is a phenomenon that has been increasing worldwide and has been associated with osteopenia. Birth before the intrauterine period of greatest incorporation of nutrients into the bone matrix results in the partial or complete loss of the optimal stage of acquisition of mineral reserves [1]. Therefore, the preterm newborn (PTNB), in addition to being metabolically immature, is more susceptible to neonatal complications, such as defective bone mineralization [2].

Globally, about 10% of births are preterm. In Brazil, the prematurity rate reaches 11.9%, which is above the world average [3]. The Northeast region is the second in the country in terms of records, only behind the Southeast region, and Ceará is one of the Northeastern states that most contribute to these numbers [4]. In preterm infants, the prevalence of osteopenia ranges from 2.5% to 50% in early postnatal life, with a tendency to increase in extreme prematurity [5, 6]. The lower the gestational age at birth, the higher the prevalence of osteopenia [7].

In recent years, the longer survival of PTNB and the parallel increase in osteopenia cases has raised clinical interest in terms of the social costs and the future quality of life of preterm infants with some degree of bone impairment. Early in life, they may present fractures and rickets [8] and, in the future, losses related to posture, mobility and muscle strength [9, 10]; shorter length, suboptimal peak bone mass [11] and higher Body Mass Index [12]. In childhood and adulthood, this scenario can contribute to poorer school performance, behavioral disorders and low self-esteem [9].

The optimization of nutrition in the postnatal period is capable of affecting not only the immediate growth and development of these newborns, but also producing effects in the long term [13]. Research on this topic is scarce in developing countries, such as Brazil, where prematurity and osteopenia are still little discussed [12, 14]. Therefore, this study was intended to address this gap by investigating the nutritional factors associated with osteopenia of prematurity in nutritional care during hospital stay.

## Methods

### Study design

This is a case–control study with preterm newborns admitted to the Neonatal Intensive Care Units of the Assis Chateaubriand Maternity School, a high-risk reference university institution, located in the capital of the State of Ceará, born from January 2018 to December 2019.

### Population

A total of 115 newborns were selected as the study population, 46 cases and 69 controls. Cases and controls included all newborns, during the study period, with gestational age less than or equal to 32 weeks at birth and length of stay greater than or equal to 8 weeks, excluding surgical newborns, those with incomplete medical records, chromosomal abnormalities, congenital anomalies, bone diseases, inborn errors of metabolism and born to diabetic mothers, if fetal macrosomia.

### Variables

Clinical data on the puerperal woman and the newborn were collected from the puerperal woman’s medical record, using a structured form with the following items: gestational data, data on the newborn at birth and data on the newborn’s clinical and nutritional follow-up. All clinical and nutritional variables were assessed by means of daily follow-up of medical prescriptions, diagnostic updates and nutritional behaviors for 8 weeks after birth.

The presence or absence of osteopenia, diagnosed through biochemical assay, was considered a dependent variable. All preterm newborns underwent biochemical screening for alkaline phosphatase and phosphorus at 3 weeks of postnatal life, being reassessed every 15 days. In any biochemical screening, PTNB with serum alkaline phosphatase levels above 900UL/l and hypophosphatemia below 4 mg/dl were diagnosed with the disease.

The optimized kinetic method at 405 nm was used to determine alkaline phosphatase, while the ultraviolet method was used to determine inorganic phosphorus. The ALP 405–AA Liquid Line–Wiener® and Phosfatemia–UV AA–Wiener® reagent kits were used. The methods were held on a biochemical analyzer, CMD800iX1 model, manufactured by Wiener Lab Group.

Independent variables of exposure included the supply of pasteurized fresh human milk with acidity greater than or equal to 4º Dornic, determined by physicochemical analysis, average intake of calories, protein, calcium, phosphorus, zinc and vitamin D, use of parenteral nutrition for more than 14 days, achievement of full enteral feeding (volume greater than or equal to 135 ml/kg/day) for more than 14 days, use of nutrient formula and nutritional supplementation of calcium, phosphorus, zinc and vitamin D.

Variables related to the puerperal women (age, education, employment link, marital status, municipality where they live, comorbidities, smoking, prenatal care and antepartum nutritional status, according to Body Mass Index by gestational age), the newborns at birth (gestational age, weight and APGAR in the first minute of life) and the period of hospital stay (time on invasive ventilatory support, duration of medication use, physical therapy intervention, comorbidities and nutritional status at term) were also collected.

Weight at birth and during hospital stay was collected with the newborn naked and in a dorsal decubitus position, and then assessed on a Balmak Baby ELP-25BB® electronic scale. During hospital stay, measurements followed the same daily weighing schedule, avoiding the bias of weight change throughout the day. The newborn’s nutritional status was assessed according to weight by corrected gestational age, using the Intergrowth-21 growth curve [15].

Regarding the nutritional therapy protocol, parenteral nutrition was installed in the first 24 h of life in newborns weighing less than 1500 g, through the peripheral route, if the osmolarity is lower than 900 mOsm/L, or the central route, if the osmolarity is higher or equal to 900 mOsm/L, per umbilical catheter (PICC). The water quota of parenteral nutrition started with 60 to 80 ml/kg/day, progressing to 10 ml/kg/day and remaining with 150 ml/kg/day, being suspended when the enteral diet supplied 2/3 of the water needs.

In the absence of abdominal distention and presence of intestinal noises and/or meconium elimination, enteral nutritional therapy was started in an early trophic way by gavage, until oral maturity, complete suction-swallow-breathing coordination and absence of respiratory discomfort, allowing the breastfeeding or oral feeding.

During hospitalization, the newborns received raw, pasteurized human milk, with or without additives, or infant formula. The type of diet offered depended on the gestational age at birth, volume, availability of human milk offered by the institution's milk bank, availability of puerperal milking and clinical evolution.

When necessary, and on medical advice, PTNBs received oral calcium, phosphorus, zinc and vitamin D supplementation. Calcium and phosphorus were supplemented in the form of tricalcium phosphate (50 mg/ml and 25 mg/ml), zinc chelate (2 mg /ml) and vitamin D at a concentration of 900 IU/ml. In parenteral nutrition, the patient receives supplementation of phosphate (1mMol), calcium (0.5 mEq/ml), amino acids (10 g/100 ml), and lipids (20 g/100 ml).

### Statistical analysis

**Fig. 1:**
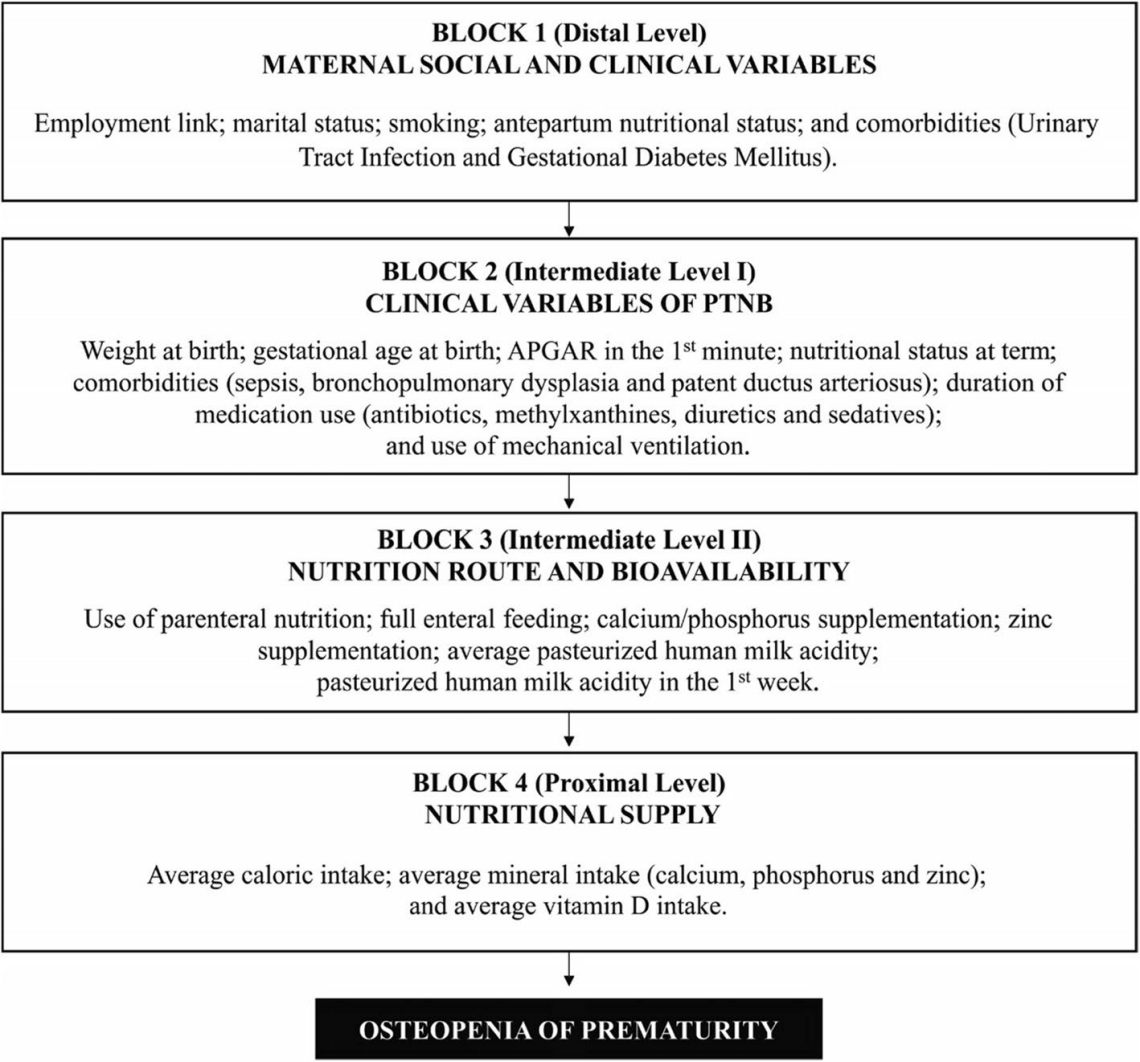
Hierarchical theoretical model of factors associated with osteopenia of prematurity

The variables were assessed using the X^2^ test and bivariate logistic regression, and then used in the multivariate analysis to investigate the factors associated with the occurrence of the disease, through logistic regression and backward stepwise method. Initially, the variables associated with the outcome in the bivariate analysis that presented a significance level ≤ 0.2 were included in the model, corresponding to their level of determination (distal, intermediate or proximal). The insertion of each independent variable in the multivariate analysis occurred according to the constructed hierarchical theoretical model, which considered four blocks of causal determination (Fig. 1).

Considering the close relationship between osteopenia of prematurity and mineral deficiency in the etiology of the disease, variables on quantitative nutritional aspects were adopted at the proximal level of causal determination, followed by variables on qualitative nutritional aspects, which are related to nutrient absorption and incorporation (intermediate level II). The characteristics of the newborn at birth and during the early postnatal life were grouped at the higher level (intermediate level I); and, at the distal level, the maternal characteristics that are more distantly related to the disease under study were included.

The variables included in each level of determination that presented *p* ≤ 0.1 were maintained in the final model and remained as an adjustment factor for the variables in the hierarchically inferior blocks. The variables that presented *p* < 0.05 in the final model as statistically significant were considered as factors associated with osteopenia of prematurity. The magnitude of the effect of each variable was estimated by calculating the *Odds ratio* and its respective 95% confidence intervals (CI). All analyses were performed using the SPSS statistical program, version 20.0.

### Ethics

This study was approved by the Research Ethics Committee of the Assis Chateaubriand Maternity School, CAAE 36,357,120.9.0000.5050, complying with the ethical criteria of Resolution 466/12, under Opinion number 4.246.798. The authors declare that all methods were carried out in accordance with relevant guidelines and regulations. The informed consent of the subjects and legal guardians was exempt by the Research Ethics Committee of Federal University of Ceará, in agreement with the Brazilian law resolution no. 466, of December 12, 2012.

## Results

Our population consisted of 115 newborns, 46 cases (40%) and 69 controls (60%). Female and male newborns constituted 53% and 47% of the study population, respectively. Most (67%) were born by means of cesarean delivery, with gestational age and average birth weight of 29 weeks (± 2.24) and 1.146 kg (± 0.36). As for the biochemical tests, during hospital stay, newborns diagnosed with osteopenia had average serum values of 1400UL/l (± 463) of alkaline phosphatase and 4.56 mg/dl (± 0.98) of phosphorus, higher than the control group (*p* < 0.001).

Maternal and study population characteristics can be seen in Tables 1 and 2. The average age of the surveyed puerperal women was 26 years (± 7.0), most have complete high school education, are not working, have a stable relationship with their partners, live in municipalities with more than 100,000 inhabitants, performed prenatal consultations, were diagnosed with urinary tract infection and pre-eclampsia during pregnancy, and presented nutritional status of excess weight in the antepartum period.

**Table 1:** Bivariate analyses related to maternal social and clinical characteristics (Block 1)

	**Total**	**Case (*n* = 46)**	**Control (*n* = 69)**	**p**
**Age**				
Average ± SD	26.4 ± 7.0	25.9 ± 6.4	26.8 ± 7.4	0.485
**Education, n (%)**				0.324^a^
Illiterate	1 (0.9)	-	1 (1.4)	
Incomplete elementary school	30 (26.1)	10 (21.7)	20 (29.0)	
Complete elementary school	14 (12.2)	9 (19.6)	5 (7.2)	
Incomplete high school	15 (13.0)	5 (10.9)	10 (14.5)	
Complete high school	44 (38.3)	16 (34.8)	28 (40.6)	
Incomplete higher education	6 (5.2)	4 (8.7)	2 (2.9)	
Complete higher education	5 (4.3)	2 (4.3)	3 (4.3)	
**Employment link, n(%)**				0.090
Without a job	65 (56.5)	22 (47.8)	43 (62.3)	
With a job	50 (43.5)	24 (52.2)	26 (37.7)	
**Marital status, n(%)**				0.166
Single	33 (28.7)	9 (19.6)	24 (34.8)	
Married	18 (15.7)	7 (15.2)	11 (15.9)	
Stable union	64 (55.7)	30 (65.2)	34 (49.3)	
**Inhabitants of the municipality, n(%)**				0.632^a^
< 10,000	1 (0.9)	-	1 (1.4)	
≥ 10,000 < 50,000	28 (24.3)	9 (19.6)	19 (27.5)	
≥ 50,000 < 100,000	19 (16.5)	8 (17.4)	11 (15.9)	
≥ 100,000	67 (58.3)	29 (63.0)	38 (55.1)	
**Health region, n(%)**				0.596^a^
Fortaleza	91 (79.1)	37 (80.4)	54 (78.3)	
Sobral	6 (5.2)	3 (6.5)	3 (4.3)	
Cariri	4 (3.5)	1 (2.2)	3 (4.3)	
Sertão Central	11 (9.6)	5 (10.9)	6 (8.7)	
Litoral Leste/Jaguaribe	3 (2.6)	-	3 (4.3)	
**Prenatal care, n(%)**				0.263^a^
Not performed	8 (7.0)	5 (10.9)	3 (4.3)	
Performed	107 (93.0)	41 (89.1)	66 (95.7)	
**Smoking, n(%)**				0.142^a^
No	107 (93.0)	45 (97.8)	62 (89.9)	
Yes	8 (7.0)	1 (2.2)	7 (10.1)	
**Antepartum nutritional status, n(%)**				0.074^a^
Low weight	13 (12.4)	5 (12.5)	8 (12.3)	
Adequate	42 (40.0)	21 (52.5)	21 (32.3)	
Excess weight	50 (47.6)	14 (35.0)	36 (55.4)	
**Comorbidities in pregnancy, n(%)**				
Urinary tract infection	45 (39.1)	21 (45.7)	24 (34.8)	0.165
Pre-eclampsia	41 (35.7)	18 (39.1)	23 (33.3)	0.330
Gestational diabetes	14 (12.2)	3 (6.5)	11 (15.9)	0.156^a^
HELLP syndrome	8 (7.0)	5 (10.9)	3 (4.3)	0.263^a^

X^2^ Test. Logistic regression

^a^ Fisher’s Exact Test

**Table 2:** Bivariate analyses related to characteristics of preterm newborns (Blocks 2, 3 and 4)

	**Total**	**Case (*n* = 46)**	**Control (*n* = 69)**	**p**
**Gender, n(%)**				0.485
Female	61 (53.0)	25 (54.3)	36 (52.2)	
Male	54 (47.0)	21 (45.7)	33 (47.8)	
**APGAR 1**^**st**^** minute, n(%)**				0.017^b^
< 7	65 (56.5)	32 (69.6)	33 (47.8)	
≥ 7	50 (43.5)	14 (30.4)	36 (52.2)	
**Gestational age at birth, n(%)**				< 0.001^b^
< 28 weeks	30 (26.1)	22 (47.8)	8 (11.6)	
≥ 28 weeks	85 (73.9)	24 (52.2)	61 (88.4)	
**Weight at birth, n(%)**				< 0.001^b^
< 1,000 g	42 (36.5)	31 (67.4)	11 (15.9)	
≥ 1,000 g	73 (63.5)	15 (32.6)	58 (84.1)	
**Clinical diagnosis, n(%)**				
Jaundice	104 (90.4)	42 (91.3)	62 (89.9)	1.000
Patent foramen ovale	75 (65.2)	29 (63.0)	46 (66.7)	0.419
Sepsis	59 (51.3)	32 (69.6)	27 (39.1)	0.001^b^
Intraventricular hemorrhage	59 (51.3)	24 (52.2)	35 (50.7)	0.515
Patent ductus arteriosus	55 (47.8)	25 (54.3)	30 (43.5)	0.170
Bronchopulmonary dysplasia	37 (32.2)	22 (47.8)	15 (21.7)	0.003^b^
Retinopathy of prematurity	36 (31.3)	15 (32.6)	21 (30.4)	0.482
**Support (Days), average ± SD**				
Non-invasive mechanical ventilation	23 ± 17.2	22 ± 17.9	23 ± 16.7	0.753
Invasive mechanical ventilation	16 ± 18.6	25 ± 21.8	10 ± 12.9	< 0.001^b^
**Physiotherapy (Days)**				
Average ± SD	49 ± 8.3	49 ± 6.7	49 ± 9.3	0.590
**Medications (Days), average ± SD**				
Antibiotics	23 ± 13.1	26 ± 12.5	21 ± 13.2	0.040^b^
Diuretics	11 ± 13.9	16 ± 15.8	7 ± 11.1	< 0.001^b^
Methylxanthines	38 ± 14.2	46 ± 10.8	33 ± 13.9	< 0.001^b^
Corticosteroids	3 ± 6.0	4 ± 6.7	2 ± 5.5	0.216
Antiulceratives	3 ± 7.7	2 ± 4.2	4 ± 9.3	0.203
Sedatives	9 ± 14.9	12 ± 15.3	8 ± 14.5	0.143
**Nutritional status at term, n(%)**				0.013^b^
Score Z ≤ -3SD	18 (15,7)	12 (26,1)	6 (8,7)	
Score Z > -3SD	97 (84,3)	34 (73,9)	63 (91,3)	
**Parenteral nutritional therapy, n(%)**				0.017^b^
≥ 14 days	73 (63.5)	35 (76.1)	38 (55.1)	
< 14 days	42 (36.5)	11 (23.9)	31 (44.9)	
**Trophic enteral feeding, n(%)**				0.255
≥ 7 days	47 (40.9)	21 (45.7)	26 (37.7)	
< 7 days	68 (59.1)	25 (54.3)	43 (62.3)	
**Full enteral feeding, n(%)**				0.001^b^
≥ 14 days	55 (47.8)	13 (28.3)	42 (60.9)	
< 14 days	60 (52.2)	33 (71.7)	27 (39.1)	
**Pasteurized fresh human milk, average ± SD**				
Total acidity (ºDornic)	3.8 ± 0.58	3.91 ± 0.55	3.77 ± 0.59	0.191
Acidity in the first week				0.025^b^
≥ 4 ºDornic, n (%)	39 (33.9)	21 (45.7)	18 (26.1)	
< 4 ºDornic, n (%)	76 (66.1)	25 (54.3)	51 (73.9)	
**Nutrient formula (FM85)**				0.202
No	41 (35.7)	19 (41.3)	22 (31.9)	
Yes	74 (64.3)	27 (58.7)	47 (68.1)	
**Supplementation, n(%)**				
Tricalcium phosphate	84 (73.0)	43 (93.5)	41 (59.4)	< 0.001^b^
Chelated zinc	44 (38.3)	13 (28.3)	31 (44.9)	0.053
Vitamin D	114 (99.1)	45 (97.8)	69 (100)	0.400
**Nutritional supply, average ± SD**				
Total energy value (kcal/kg)	88.9 ± 20.8	84.1 ± 9.3	92.3 ± 25.4	0.013^b^
Protein (g/kg)	2.9 ± 0.3	2.92 ± 0.34	2.94 ± 0.27	0.629
Calcium (mg/kg)	169.9 ± 43.9	182.7 ± 47.5	161.5 ± 39.5	0.013^b^
Phosphorus (mg/kg)	89.4 ± 28.4	98.3 ± 31.3	83.5 ± 24.8	0.008^b^
Zinc (mcg/kg)	659 ± 348	554 ± 326	729 ± 347	0.010^b^
Vitamin D (UI/dia)	485 ± 176	410 ± 160	536 ± 169	< 0.001^b^
Average calcium/phosphorus ratio	1.95 ± 0.25	1.92 ± 0.25	1.96 ± 0.25	0.367

X^2^ Test. Logistic regression

^b^ *p*-value significant

Regarding the newborns, the results of the bivariate analysis show that osteopenia is associated with worse conditions at birth, such as extreme prematurity [OR 2.59 (CI 1.74–3.88); *p* < 0.001], extremely low birth weight [OR 3.59 (CI 2.21–5.84); *p* < 0.001] and lower APGAR index in the first minute of life [OR 1.76 (CI 1.06–2.92); *p* = 0.017]; and, throughout hospital stay, it is associated with the use of invasive ventilatory support for a prolonged time [OR 1.05 (CI 1.03–1.08), *p* < 0.001], presence of sepsis [OR 1.64 (CI 1.19–2.25); *p* = 0.001] and bronchopulmonary dysplasia [OR 1.71 (CI 1.13–2.59); *p* = 0.003].

As for the nutritional aspects, in the bivariate analysis, osteopenia is associated with the intake of pasteurized fresh human milk with acidity greater than or equal to 4º Dornic [OR 1.45 (CI 1.001–2.11); *p* = 0.025], the use of parenteral nutrition [OR 1.42 (CI 1.07–1.88); *p* = 0.017] and full enteral feeding for more than 14 days [OR 0.59 (CI 0.43–0.81); *p* = 0.001]. Nutritional supply also showed a difference between cases and controls, where the higher caloric intake [OR 0.95 (CI 0.92–0.99); *p* = 0.013] and the higher vitamin D intake [OR 0.99 (CI 0.99–0.99); *p* < 0.001] were protective factors, while the higher calcium intake [OR 1.01 (CI 1.00–1.02); *p* = 0.013] and the higher phosphorus intake [OR 1.02 (CI 1.00–1.03); *p* = 0.008] were risk factors.

After multivariate analysis and adjustments, the variables that remained in the final model of causal determination can be seen in Table 3. Regarding single puerperal women, the presence of a partner was a protective factor and decreased the chances of developing osteopenia [OR 0.10 (CI 0.02–0.59); *p* = 0.038], as well as the use of sedatives in the newborn for a shorter time [OR 0.89 (CI 0.83–0.98); *p* = 0.010]. The probability of developing the disease was higher in newborns with extremely low birth weight [OR 5.49 (CI 1.20–25.1); *p* = 0.028], who developed sepsis during hospital stay [OR 5.71 (CI 1.35–24.2); *p* = 0.018] and who used, for a long time, invasive ventilatory support [OR 1.09 (CI 1.03–1.18); *p* = 0.007].

**Table 3:** Final model of multivariate logistic regression of factors associated with osteopenia of prematurity

	**Not-adjusted Model**		**Adjusted Model**	
	**OR (95% CI)**	**p**	**OR (95% CI)**	**p**
**BLOCK 1: Maternal social and clinical variables**				
Marital status		0.166		0.038^b^
Single	1		1	
Married	0.43 (0.17–1.06)		0.10 (0.02–0.59)	
Stable union	0.72 (0.25–2.09)		0.72 (0.11–4.79)	
Smoking		0.142		0.171
Yes	1		1	
No	0.66 (0.49–0.90)		21.8 (0.27–17.88)	
Antepartum nutritional status		0.074		0.131
Adequate	1		1	
Low weight	3.86 (0.94–15.7)		2.71 (0.30–24.5)	
Excess weight	2.57 (1.08–6.10)		6.72 (1.33–34.0)	
Comorbidities in pregnancy				
Urinary tract infection	1.21 (0.87–1.67)	0.165	0.56 (0.14–2.21)	0.411
**BLOCK 2: Clinical variables of the preterm newborn**				
Weight at birth		< 0.001		0.028^b^
≥ 1,000 g	1		1	
< 1,000 g	3,59 (2.21–5.84)		5.49 (1.20–25.1)	
APGAR 1^st^ minute		0.017		0.273
≥ 7	1		1	
< 7	1.76 (1.06–2.92)		2.22 (0.53–9.26)	
Ventilatory support				
Mechanical ventilation	1.05 (1.03–1.08)	< 0.001	1.09 (1.03–1.18)	0.007^b^
Clinical diagnosis				
Sepsis	1.64 (1.19–2.25)	0.001	5.71 (1.35–24.2)	0.018^b^
Medications (Duration)				
Methylxanthines	1.09 (1.05–1.13)	< 0.001	1.06 (0.99–1.13)	0.060
Sedatives	1.02 (0.99–1.05)	0.143	0.89 (0.83–0.98)	0.010^b^
**BLOCK 3: Nutrition routes and bioavailability**				
Acidity in the first week		0.025		0.035^b^
< 4 ºDornic	1		1	
≥ 4 ºDornic	1.45(1.001–2.11)		5.36 (1.13–25.5)	
**BLOCK 4: Nutritional supply**				
Calcium (mg/kg)	1.01 (1.00–1.02)	0.013	1.05 (1.006–1.1)	0.025^b^
Phosphorus (mg/kg)	1.02 (1.00–1.03)	0.008	0.95 (0.89–1.01)	0.091

OR not-adjusted: Bivariate analysis

OR adjusted: adjustment by variables internal to the block and by previous blocks

Final model adjustment: -2 Log LR = 71.007; R^2^ = 0.699; *p* < 0.001

^b^ *p*-value significant

As for the nutritional factors, the association of the disease with the human milk acidity and the calcium intake was maintained. Newborns who drink more acidic pasteurized fresh human milk in the early postnatal life are 5.36 times more likely to develop osteopenia (*p* = 0.035). The higher calcium intake, compared to the control group, also increased the probability of disease occurrence [OR 1.05 (CI 1.006–1.1); *p* = 0.025] (Table 3).

## Discussion

This study identified that osteopenia of prematurity is associated with nutritional factors, such as human milk acidity and high calcium intake. Extremely low birth weight, presence of sepsis, invasive ventilatory support and use of sedatives are important factors to be considered at birth and during hospital stay, and the presence of a partner is a protective maternal social component associated with the disease.

The low solubility and precipitation of nutrients is a problem reported in studies that address osteopenia of prematurity, especially in the face of prolonged use of parenteral nutrition as the sole or main dietary source [12]. Therefore, along with parenteral nutrition, early enteral nutrition is recommended [16].

It is known that some nutrients are increased in the milk of puerperal mothers of preterm newborns and the calcium and phosphorus contents vary between 208 and 216 mg/L, and 95 and 143 mg/L, respectively, but they do not seem to be sufficient to meet the recommendations of the RNPT [17]. In addition, in neonatal health units, much of the human milk offered comes from the Human Milk Bank (HMB) [18]. As most of the donations are from postpartum mothers of term newborns and human milk undergoes pasteurization processes, the nutritional content also does not seem to be sufficient to meet the needs of these newborns. A lower nutritional supply results in low serum concentrations and lower mineral incorporation into the bone matrix [19].

A dietary alternative, infant formula specifically for preterm infants, is richer in calcium and phosphorus than human milk. However, calcium bioavailability ranges from 35 to 60% of intake. Therefore, in relation to infant formula, the intake of human milk should be promoted and encouraged [20].

In PTNB, the mother's own milk should be used and in case the mother's milk is not available then the donor breast milk can be used instead [21]. After milking, some bacteria are able to ferment the lactose present in milk and produce lactic acid, which, in addition to increasing acidity, raises osmolarity and reduces the bioavailability of calcium and phosphorus, leading to precipitation and insolubilization of minerals. Therefore, even though they are quantitatively present and susceptible to detection, minerals have their bioavailability reduced [22].

The longer the period between milking and provision, the greater the amount of lactic acid produced and, consequently, the lower the bioavailability of calcium and phosphorus in milked human milk [22]. Considering this qualitative aspect becomes relevant in nutritional care, whether during the provision of raw fresh human milk, which should be offered, preferably, right after milking, at the bedside [23]; or during the provision of pasteurized fresh human milk, whose result in our study encourages acidity lower than 4º Dornic in the first week on enteral diet.

Regarding the quantitative nutritional supply, this study showed data contradictory to the literature. One of the most pointed etiological factors for osteopenia is the inadequate intake of calcium and phosphorus [1]. For this reason, since the 1990s, attention to early supply of minerals and supplementation of calcium and phosphorus has been recommended for newborns at risk [24], with the objective of providing postnatal nutritional support similar to that provided in the intrauterine environment, allowing for adequate growth and development [7].

In the current study, although both groups had mineral intake within the adequate ranges, the higher calcium intake increased the chance of developing osteopenia, even after adjustments. On the one hand, this result represents the need for reassessment and discussion of current recommendations for mineral nutritional intake in preterm newborns in the early postnatal period, as highlighted by Avila-Alvarez et al [25]. On the other hand, Sharp and Simmer [26], in 2003, already reported the importance of considering the bioavailability of supplemented minerals.

In this study, newborns with osteopenia received mineral supplementation in a greater proportion and for a longer time, compared to their controls [21 days (± 11.6) vs. 13 days (± 12.9), *p* = 0.002]; and, in the bivariate analysis, supplementation was also associated with the disease. The tricalcium phosphate form, in the calcium to phosphorus ratio of 2:1, adopted in the research institution, has a reported bioavailability of approximately 38% [27]. In addition, inorganic phosphate salts readily precipitate when combined with calcium supplementation, reducing the bioavailability of both minerals [28]. Therefore, even with a quantitatively adequate supply, after supplementation, and even higher than controls, these minerals may not have been effectively absorbed and used by the surveyed newborns.

This study also demonstrated the difficulty of providing nutritional support according to the recommendations. According to the findings, this nutritional inadequacy seems to be related to the lower volume of diet offered, which also reflects a lower mineral supply and contributes to the occurrence of osteopenia. As shown by Chen et al. [16], reduced enteral diet volume is a risk factor for osteopenia. The volume of human milk provided early in postnatal life is positively associated with bone mineral content [21].

Growth can also be considered an important indicator of bone mineralization [29]. Monitoring food intake and growth curves contribute to the optimization of extrauterine growth and development [21]. Even without association with osteopenia after adjustments, in this study, 15.7% of newborns had a nutritional diagnosis of malnutrition at term age, showing the importance of monitoring nutritional status in dietary choices during hospitalization and after hospital discharge.

As for the maternal aspects, the results found show that the chances of developing osteopenia are lower in newborns conceived by married women. Puerperal women who gave birth to preterm babies experience difficulties, such as, for example, stress (due to prematurity and low birth weight), separation from the binomial, discomfort with the environment of the neonatal unit, feeling of doubt, fear, sadness and anguish. All these situations undermine the family coexistence and dynamics, in addition to interfering with the production and maintenance of lactation and the desire to breastfeed and/or express human milk [30]. Therefore, they need to be welcomed and provided with a well-structured support network, as their isolation in the postpartum period becomes common.

This support is structured in the care at home, with the other children, in the shared information and social experiences, so that the puerperal woman can step away from external reality and engage in the care of the newborn [22]. In light of the foregoing, it should be highlighted the importance of encouraging breastfeeding and public policies for the care of women’s health from prenatal to outpatient preterm follow-up [31], as well as the need for evidence-based guidelines for standardized management and nutritional practice in osteopenia, especially in the face of other risk situations at birth and during hospital stay [32].

Regarding birth-related risk factors, it is observed that PTNBs with osteopenia have lower gestational age, birth weight and APGAR in the first minute of life [25]. In this study, extremely low birth weight was one of the factors that most increased the chance of osteopenia. Literature shows that the incidence of the disease can reach 60% in this condition at birth [8]. Preterm newborns have immature physiology, low food tolerance and increased risk of clinical morbidities. Restricted intrauterine growth and compromised birth weight contribute to the development of osteopenia, since these factors reflect a worse nutritional reserve, already compromised in prematurity, impairing the achievement of adequate caloric and mineral intake, the recovery of the postnatal nutritional status, the growth and the mineral incorporation into the bone matrix [33].

PTNB also have an intestinal microbiome profile that makes them more vulnerable to infections and metabolic complications [34]. As with extremely low weight, the presence of sepsis is associated with an increased probability of developing osteopenia. In situations of sepsis, antibiotic treatment impacts the intestinal flora, impairing the integrity of the mucosa, the absorptive capacity of the small intestine, as well as the mineral solubility and absorption [35]. In addition, the presence of sepsis is associated with clinical instability and restricted postnatal weight gain because it causes total or partial intolerance to enteral feeding, making a nutritional approach more difficult [1].

Another important issue is that the hypophosphatemia present in osteopenia causes muscle weakness, impairing exercise performance and leading to immobility [25]. In more severe conditions, the use of sedatives and ventilatory support during hospital stay increases the period of immobilization in bed and entails minimal handling, contributing to increased bone resorption and demineralization, increasing the chances of developing osteopenia [36], as found in the current study. Since bone is an organ, its formation depends on its function. Stimulation increases bone formation and leptin markers, attenuating natural postnatal bone decline, and helps ensure adequate bone mineral content and muscle development [5].

Finally, the main limitation of the study is the absence of imaging tests, considered the gold standard in the assessment of osteopenia. Despite the widespread recognition of the disease, there is still no unified approach to diagnosis. Different markers are studied in the screening for osteopenia, although there is no agreement regarding their clinical use [37]. Alkaline phosphatase and phosphorus are widely used in the investigation of osteopenia by 92% and 77% of neonatologists in health services, respectively [38].

The study showed an incidence of 40% of the disease, which is within the range reported in the literature. Although it is suggested that early diagnosis based on biochemical tests may overestimate cases of osteopenia, our result corroborates a recent study by Angelika et al. [39], which found 43% of newborns with the disease, diagnosed by imaging tests, highlighting its importance as an incident comorbidity in preterm newborns, regardless of the screening method.

Another limitation is based on the type of study performed. As data collection took place retrospectively, it was not possible to assess relevant variables for the study, such as food intake and nutritional deficiencies of puerperal women during pregnancy. However, as far as the authors are aware, this is the first study that assessed the human milk acidity as a qualitative aspect associated with osteopenia of prematurity. In addition, a monocentric study was carried out, where the newborns followed the same nutritional protocol and were followed-up daily from birth to 8 weeks after birth.

## Conclusions

This study brings up important contributions in terms of directing strategies for prevention and control of osteopenia in preterm newborns. It is concluded that the main nutritional factors associated with the disease were acidity and high calcium intake. It launched a relevant theme for further studies on the use and distribution of human milk with lower acidity at the beginning of the introduction of enteral feeding, capable of decreasing the chances of disease occurrence. The high calcium intake and the increased chance of osteopenia constituted an unexpected result, which confirms the need for studies on mineral nutritional recommendation and supplementation. It is reiterated that attention must be reinforced in other risk situations, such as newborns conceived by single women, with compromised birth weight, who present infectious complications during hospital stay and undergo long periods of sedation and ventilatory support.

## Data Availability

The datasets used and/or analysed during the current study are available from the corresponding author on reasonable request.

## Abbreviation

PTNB: Preterm newborn
